# Structural Prediction and Characterization of *Canavalia grandiflora* (ConGF) Lectin Complexed with MMP1: Unveiling the Antiglioma Potential of Legume Lectins

**DOI:** 10.3390/molecules27207089

**Published:** 2022-10-20

**Authors:** Rodrigo Bainy Leal, Josiane Mann, Vanir Reis Pinto-Junior, Messias Vital Oliveira, Vinicius Jose Silva Osterne, Ingrid Alessandra Victoria Wolin, Ana Paula Machado Nascimento, Priscilla Gomes Welter, Valeria Maria Sousa Ferreira, Alice Araujo Silva, Rodrigo Lopes Seeger, Kyria Santiago Nascimento, Benildo Sousa Cavada

**Affiliations:** 1 Departamento de Bioquímica, Universidade Federal de Santa Catarina (UFSC), Florianópolis 88040-900, SC, Brazil; 2 Departamento de Física, Universidade Federal do Ceará (UFC), Fortaleza 60440-970, CE, Brazil; 3 Departamento de Bioquímica e Biologia Molecular, Universidade Federal do Ceará (UFC), Fortaleza 60440-970, CE, Brazil; 4 Department of Biotechnology, Ghent University, 9000 Ghent, Belgium

**Keywords:** glioblastoma, lectin, *Canavalia grandiflora*, matrix metalloproteinase 1

## Abstract

A glioblastoma (GBM) is a highly malignant primary brain tumor with a poor prognosis because of its invasiveness and high resistance to current therapies. In GBMs, abnormal glycosylation patterns are associated with malignancy, which allows for the use of lectins as tools for recognition and therapy. More specifically, lectins can interact with glycan structures found on the malignant cell surface. In this context, the present work aimed to investigate the antiglioma potential of ConGF, a lectin purified from *Canavalia grandiflora* seeds, against C6 cells. The treatment of C6 cells with ConGF impaired the mitochondrial transmembrane potential, reduced cell viability, and induced morphological changes. ConGF also induced massive autophagy, as evaluated by acridine orange (AO) staining and LC3AB-II expression, but without prominent propidium iodide (PI) labeling. The mechanism of action appears to involve the carbohydrate-binding capacity of ConGF, and in silico studies suggested that the lectin can interact with the glycan structures of matrix metalloproteinase 1 (MMP1), a prominent protein found in malignant cells, likely explaining the observed effects.

## 1. Introduction

Primary central nervous system (CNS) tumors have been the subject of intense study, mainly by their lethality and poor treatment responses. In the last decade, the World Health Organization (WHO) has improved the classification of tumors of the CNS, publishing the latest guidelines in 2021. Diffusely infiltrating gliomas are the most common tumors to arise within the brain parenchyma of adults, accounting for more than 70% of all such neoplasms [1,2]. In this context, glioblastoma (GBM) emerges as a highly infiltrative tumor, displaying necrosis and/or microvascular proliferation that represents half of all gliomas diagnosed [1,2]. Therefore, a glioblastoma (GBM) is considered the most aggressive malignant brain tumor because patients have a high recurrence after undergoing current treatments with a median survival of 14.2 months [3,4].

These statistics call for the intensified search for new therapeutic agents to treat cancer, particularly gliomas, considering the rates of incidence and mortality on a global scale [5]. Owing to the demand for specific and effective alternatives against this set of diseases, biomolecules appear as a new possibility. In particular, aberrant glycan structures in the glycoconjugates present in cancer cells offer one such alternative, namely lectins. Lectins comprise a class of proteins capable of interacting specifically and reversibly with carbohydrates. As such, they have been studied as tools in tumor recognition based on their ability to differentiate between benign masses and malignant tumors owing to the type and degree of glycosylation [6]. In addition to the recognition of glycans in cancer cells, several lectins also demonstrate direct antiproliferative activity. This effect results from the binding of lectins with glycans in the cell membrane, eliciting processes such as agglutination or aggregation concomitantly, or not, with the induction of apoptosis, autophagy, and/or necrosis [7].

Studies have shown the antiproliferative effect of lectins from legumes belonging to the Diocleinae subtribe, such as *Canavalia ensiformis* (ConA), *C. brasiliensis* (ConBr), *C. bonariensis* (CaBo), *C. virosa* (ConV), *Dioclea lasiophylla* (DlyL), *D. violacea* (DVL), and *D. lasiocarpa* (DLL) in tumor cell lines [8]. All these lectins could induce a significant reduction in the cell viability in C6 and U87 glioma cells. Some of these lectins were able to alter the mitochondrial membrane potential, cell migration, and induce morphological changes in cells. A colorimetric and biochemical analysis indicated cell death by autophagy, apoptosis, and/or necrosis via mitochondrial modulation and metalloproteinases [9,10,11,12]. Despite all these observations, specific molecular targets for legume lectins that trigger tumor cytotoxicity have not been identified. Aberrant glycosylation is related to cancer cell survival, the induction of angiogenesis, the upregulation of vascular endothelial growth factor A (VEGFA), and changes in the expression and activity of matrix metalloproteinases (MMPs), as well as cancer cell invasion and metastasis [13,14].

ConGF is a lectin from *Canavalia grandiflora* seeds, and, similar to ConA, it belongs to the subtribe Diocleinae of the legume family. Like all ConA-like lectins, it is a lectin with specificity for mannose and glucose. It presents a protomer with a molecular weight between 25–30 kDa capable of forming a homotetramer in solution, and its primary and three-dimensional structures have been determined in previous studies [8,15]. ConGF has demonstrated interesting biological effects, such as vasorelaxant activity, eliciting 25% vasorelaxation in pre-contracted endothelialized aortic rings, and an anti-inflammatory and analgesic effect. ConGF inhibited inflammatory effects generated by carrageenan in paw edema assays, additionally blocking cytokine release and neutrophil migration and reducing hypernociception. All these effects involve the participation of the carbohydrate-recognition domain (CRD) and a mechanism involving nitric oxide [15,16].

The present work aims to describe the antiglioma potential of ConGF and to point a possible target engaged in the cytotoxic response induced by the lectin. Thus, using a C6 glioma cell culture model and molecular docking, we suggest that the modulation of metalloproteinase 1 (MMP1), via a lectin glycan interaction, may take part in the glioma cell death mechanism triggered by ConGF.

## 2. Results and Discussion

**Figure 1:**
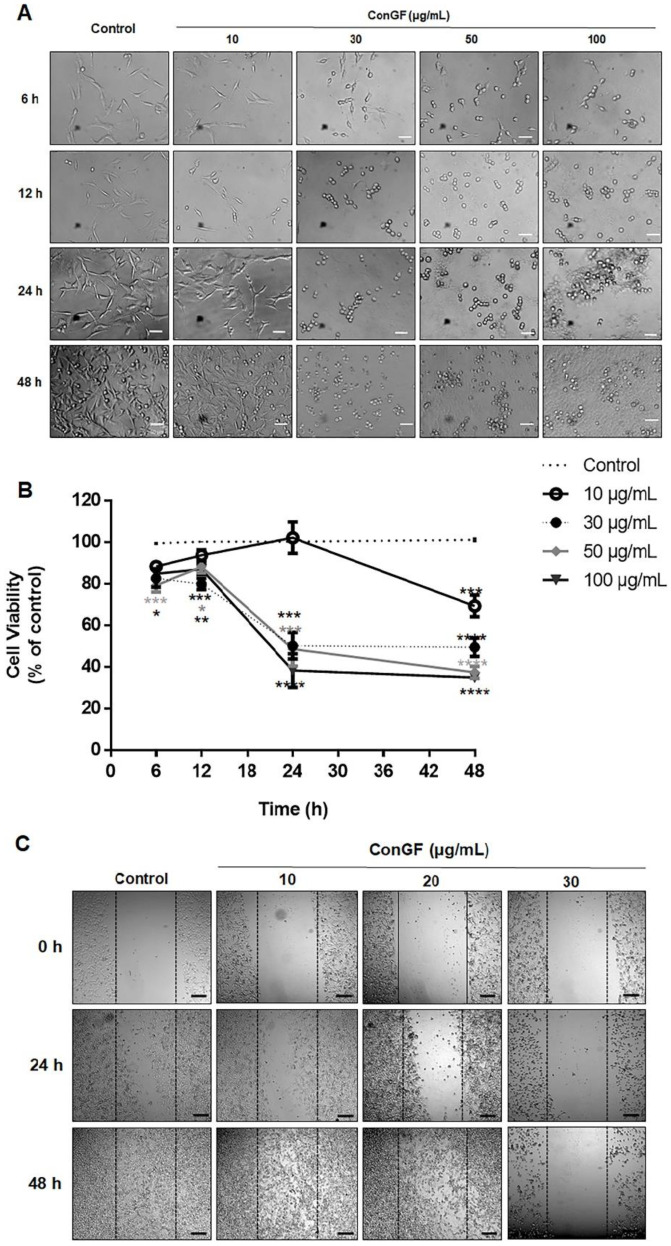
**ConGF induces morphological alteration and decreases cell viability.** C6 glioma cells were exposed for 6, 12, 24, and 48 h to vehicle (HEPES–saline buffer; control) or lectin ConGF (10, 30, 50, and 100 µg/mL). Thereafter, cell morphology and viability were performed. (**A**) Representative images from four independent experiments performed in triplicate, showing cell morphology, as evaluated by optical microscopy, in response to ConGF (bars represent 100 μm). (**B**) Cell viability evaluation after ConGF treatment, assessed by MTT assay. (**C**) Microphotographs of the wound healing at 0, 24, and 48 h after creating the wounds to evaluate cell migration (bars represent 200 μm). The results of the MTT assay were expressed as a percentage relative to the control (dotted line). Data are presented as mean ± standard error of mean (SEM). N = 4. * *p* < 0.05; ** *p* < 0.01; *** *p* < 0.001; and **** *p* < 0.0001 indicate the statistical difference compared to the control (dotted line) by one-way ANOVA, followed by Bonferroni’s post hoc test.

The cytotoxic potential of ConGF on C6 cells was investigated by an MTT assay. For this purpose, the cells were treated with the vehicle (control; HEPES/saline) or ConGF at concentrations of 10, 30, 50, or 100 μg/mL for 6, 12, 24, or 48 h. Figure 1B shows that the cell treatment with 30, 50, or 100 μg/mL of ConGF for 6, 12, 24, or 48 h caused a significant reduction in cell viability. In contrast, 10 μg/mL of ConGF had no significant effect on cell viability, except during the incubation period of 48 h. A cell analysis by light microscopy showed that ConGF induced a morphological alteration of the cells from the concentration of 30 μg/mL in all the evaluated periods (6–48 h). The main cell alteration was a change from the fusiform morphology to cells with a spherical shape that could be detached easily from the plate (Figure 1A). Moreover, the cell migration (24–48 h) was inhibited by ConGF at 30 μg/mL (Figure 1C), but not by lower concentrations, such as 10 or 20 μg/mL.

**Figure 2:**
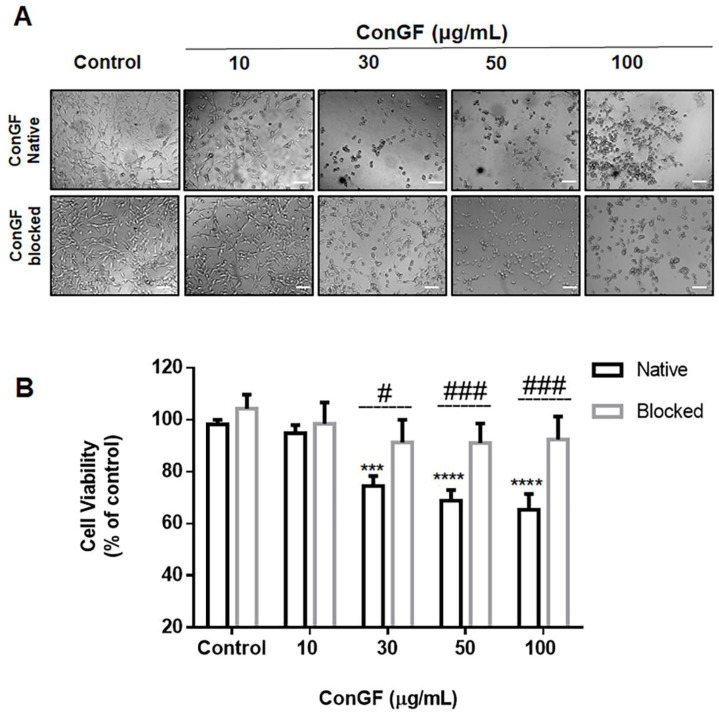
**Effect of ConGF on C6 glioma cell viability is dependent of CRD.** C6 glioma cells were treated with vehicle (HEPES; control), native ConGF (10, 30, 50, or 100 μg/mL), or ConGF blocked with α-methyl-d-mannoside for 24 h. (**A**) Representative images of cell morphology captured by inverted NIKON eclipse T2000-U microscope (10× magnification) treated with native and blocked lectin. (**B**) Cell viability assessed by the MTT assay. Values are represented as mean ± SEM of three independent experiments performed in triplicate. *** *p* < 0.001 and **** *p* < 0.0001 when compared to the vehicle-treated group (control) and ^#^  *p* < 0.05 and ^###^ *p* < 0.001 when compared to the native lectin-treated group.

In order to characterize the role of glycan binding on the lectin’s antitumor effects, ConGF was subjected to a blockade of its CRD by preincubation with α-methyl-d-mannoside. Figure 2 shows the results of the cell viability and morphology 24 h after treatment with either the native ConGF or CRD blockage. A reduction in the cell viability was only induced by treatment with the native ConGF, while the CRD blockage lectin produced no significant effects on the cell viability (Figure 2B). Moreover, the CRD-blocked lectin minimized changes to the cell morphology (Figure 2A), otherwise seen in the treatment with the native ConGF (30–100 μg/mL).

**Figure 3:**
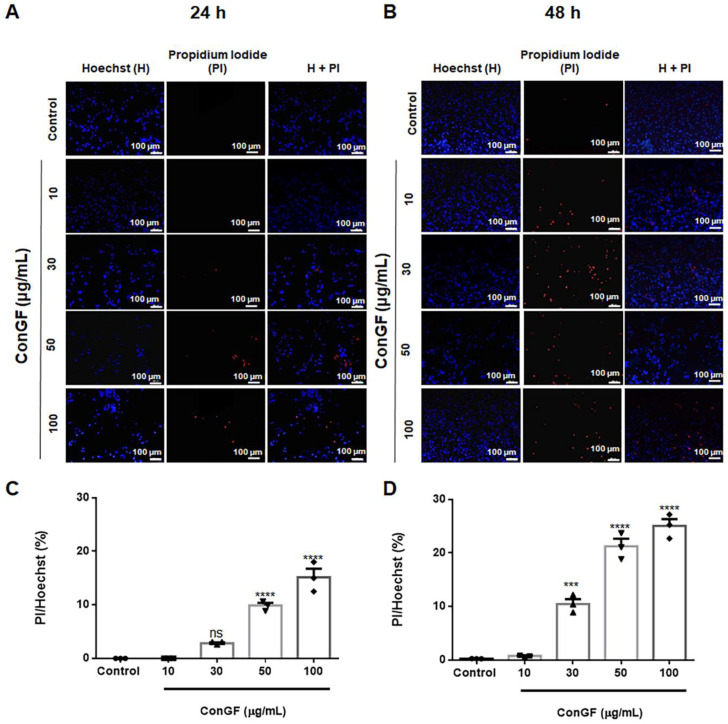
**Evaluation of C6 glioma cell membrane injury in response to ConGF treatment.** C6 glioma cells were incubated with vehicle (control) or ConGF (10, 30, 50, or 100 μg/mL) for 24 or 48 h. The cells were stained with Hoechst and propidium iodide (PI). (**A**,**B**) are representative images of Hoechst and PI staining in response to ConGF treatment for 24 and 48 h, respectively. (**C**,**D**) Quantification of PI staining in response to ConGF treatment for 24 and 48 h, respectively. Data were expressed as a percentage and the values are presented as mean ± SEM. (The scale bar represents 100 μm). For all analyses, four independent experiments were performed. One-way ANOVA followed by the Bonferroni post-hoc test. *** *p* < 0.001 and **** *p* < 0.0001, as compared to control. ns = no significative.

To characterize the type of cell death, double labeling with Hoechst and propidium iodide (PI) was performed. The captured images were analyzed using ImageJ software (U.S National Institutes of Health, Bethesda, MD, USA) and the fluorescence was quantified. The results showed that the ratio between the mean fluorescence of the PI-positive cells by the mean fluorescence of the Hoechst-positive cells (PI/Hoechst) was not remarkable for ConGF at 10 or 30 μg/mL for 24 h as compared to the control (Figure 3A,C). Moreover, the ConGF treatment at 30 μg/mL for 48 h produced only a 10% increase in the PI/Hoechst ratio (Figure 3B,D). Even treatment at the highest concentrations (50 and 100 μg/mL) for 24 or 48 h only produced an increment around 20% (Figure 3). Taken together, these results suggest that the lectin did not produce cell membrane disruption as a primary cell death mechanism.

**Figure 4:**
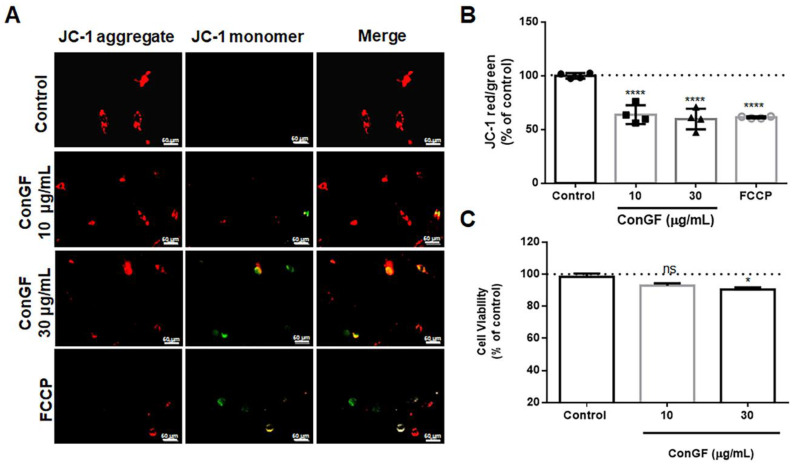
**ConGF alters mitochondrial membrane potential.** C6 glioma cells were incubated with vehicle (control) or ConGF (10 or 30 μg/mL) for 3 h. Carbonyl cyanide p-trifluoromethoxy phenylhydrazone (FCCP), a mitochondrial uncoupler, was used as positive control to fluorescence emission. (**A**) Illustrative images from cells stained with JC-1. (**B**) shows mitochondrial potential evaluated by JC-1 after 3 h of treatment. (**C**) shows cell viability measured by the MTT assay after ConGF treatment for 3 h. The data are expressed as a percentage of control, and the values are presented as mean ± SEM of four independent experiments performed in triplicate. * *p* < 0.05, **** *p* < 0.0001, as compared to control. ns = no significative.

To assess the possible effects of ConGF on the mitochondrial membrane potential (ΔΨm) of C6 cells, we used the JC-1 dye, a cationic carbocyanine dye that accumulates in mitochondria (Figure 4). We performed this assay because a loss of ΔΨm may be an early event in the apoptotic process [17]. The evaluation was carried out after a 3 h incubation when the cell viability showed no substantial changes (Figure 4C). JC-1 indicates the mitochondrial polarization by changing its fluorescence from green (525 nm) to red (590 nm) in a potential-sensitive manner. The functional mitochondria have a highly negative ΔΨm, and JC-1 penetrates and accumulates, creating aggregates that emit a red color. In the monomeric form, JC-1 emits a green color, indicating a decrease in the ΔΨm. The cells treated with ConGF for 3 h (Figure 4A,B) displayed a significantly lower ΔΨm, expressed as the ratio of red/green fluorescence, by approximately 45% compared to the control cells. Carbonyl cyanide-4 (trifluoromethoxy) phenylhydrazone (FCCP), known as an uncoupling agent of the mitochondrial electron transport chain, was used as the positive control and also showed a lower ratio of the emitted fluorescence. Although the ΔΨm was impaired after 3 h of treatment with ConGF (10–30 μg/mL), only a very small change in the response to ConGF at 30 μg/mL was shown by the MTT assay during this early period (Figure 4C). Therefore, it can be concluded that the lectin can trigger a mitochondrial alteration in an early period.

**Figure 5:**
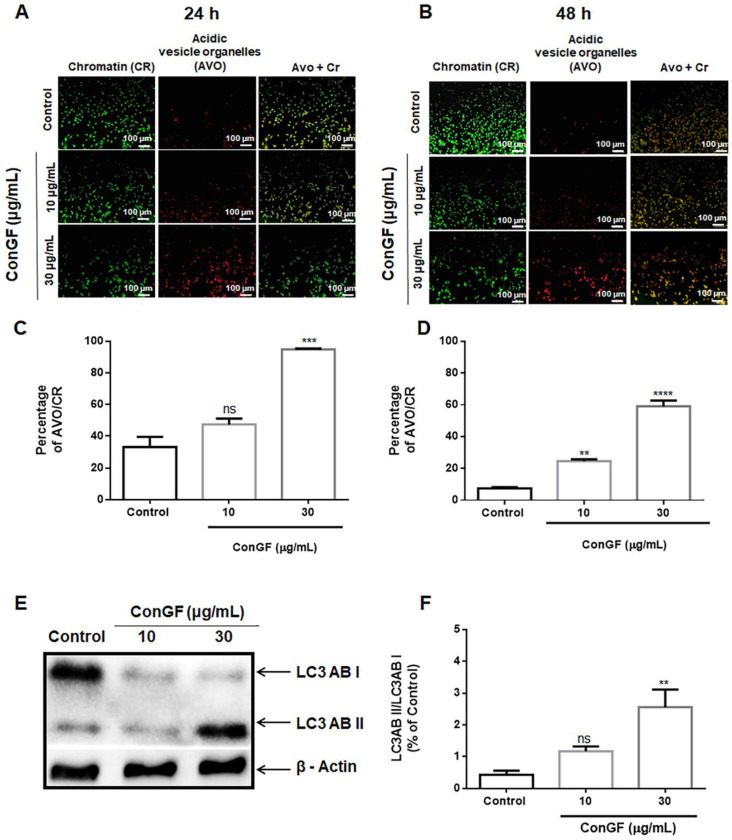
**ConGF induces autophagy in C6 glioma cells**. (**A**,**B**) show representative images of acridine orange staining of C6 glioma cells after incubation with vehicle (control) or ConGF (10 or 30 μg/mL) for 24 and 48 h, respectively. (**C**,**D**) Quantification of AO staining in response to ConGF. Chromatin (CR) and acidic vesicle organelles (AVO) were detected in green and red channels, respectively. The data are presented as percentage of AVO/Chr, and data are expressed as mean ± SEM. (The scale bar represents 100 μm). (**E**) Western-blot analysis of LC3-I and LC3-II, in C6 cells treated for 12 h with vehicle (control) or ConGF (10 or 30 μg/mL). (**F**) Quantification of the band intensity was expressed as ratio of LC3-II/LC3-I. ** *p* < 0.01, *** *p* < 0.001, and **** *p* < 0.0001, as compared to control. ns = no significative.

Because autophagy is recognized as an important mechanism for tumor cell death in response to lectins, we turned to fluorescent dyes, such as acridine orange (AO), which is used to label the acidic vesicular organelles (AVOs) present during autophagy [18]. In this way, the cells undergoing autophagy can be detected under a fluorescence microscope. Because ConGF promotes the death of C6 glioma cells, which may be related to autophagy, we investigated whether treatment with ConGF could induce an increase in the autophagic process. To make this determination, the cells were again treated with ConGF at 10 and 30 μg/mL for 24 or 48 h, followed, this time, by staining with AO and observation under an inverted fluorescence microscope. Figure 5A,C show that treatment with ConGF at 30 μg/mL for 24 h and treatment with ConGF at 10 and 30 μg/mL for 48 h (Figure 5B,D) induced a significant increase in the AVO labeling in the C6 cells. Hence, the effect of ConGF at 30 μg/mL was higher than 60%, and it was reinforced by the demonstration of a significant increment of the autophagy marker LC3ABII (Figure 5E,F), as evaluated after a 12 h incubation with the lectin.

Previously, ConA was found to induce antitumor activity through autophagy in different types of cancer, such as hepatoma [19], cervical cancer [20], and glioblastoma [9,10,11]. Among different proposed mechanisms is the interaction between the lectin and glycans associated with proteins present on the plasma membrane, triggering intracellular signaling processes [12,21]. Hence, intracellular targets can be modulated through these processes, including mitochondria, with the generation of the reactive oxygen species (ROS) and cytochrome C release, followed by the modulation of caspases and autophagy [12,21]. Moreover, through the modulation of Ras/ERK1/2, PI3K/Akt, mTORC1, and NF-kB, plant lectins may trigger these cell fates, especially autophagic cell death [10,11,12,21].

In glioblastoma cells, ConA promoted autophagy through the simultaneous negative regulation of MEK/ERK and PI3K/Akt [20]. In C6 glioma cells, studies by our group showed that ConA-like lectins induced autophagy and inhibited cell migration [8,12,20,22]. In the present study, we bring one more lectin member of the Canavalia genus, ConGF, and prove its ability to inhibit C6 glioma cell viability and also promote intense autophagy without damaging the cell membrane. Furthermore, even at low concentrations, we showed that ConGF could impair the mitochondrial membrane potential. Despite verifying these antiglioma effects produced by leguminous lectins of the Canavalia and Dioclea genera, very little is known about the primary molecular targets that could interact with these lectins and bring about the observable effects.

Matrix metalloproteinases are glycoproteins that have been evaluated in various gliomas [23,24]. MMPs-13, -17, -19, and -24 are expressed in all cell lines studied, while MMP-1, -2, -7, -9, -11, -12, -14, -15, and -25 seem to be correlated with tumor grade [24]. Several studies have shown an increased expression of MMP-1 in glioblastoma when compared with astrocytomas of a low malignancy grade, or with normal brain tissue [23,25,26,27]. Some studies have pointed out the ability of the ConA lectin to modulate the activity and expression of matrix metalloproteinases [28]. In tumor cells, ConA was able to stimulate the expression and activity of membrane metalloproteinase MT1-MMP (MMP-14), as well as MMP-2, in HeLa cells [20] and in the glioma lineage U87 [29,30]. In addition, U87 glioblastoma cells showed the upregulation of autophagy-associated proteins, including BNIP3, ATG12, and ATG13, when treated with ConA by an MT1-MMP-dependent mechanism [9,31]. Collagenase MMP-1 is a glycoprotein that has a traditional role in the cleavage of the primary substrates of the extracellular matrix (ECM), activating latent forms of bioactive molecules, which are responsible for pro-metastatic and pro-oncogenic signaling. The expression of MMP-1 is regulated by EGFR because it was shown that the receptor inhibitor AG1478 led to the suppression of MMP-1 levels and decreased tumor invasion [32]. Furthermore, MMP-1 can cleave the PAR1 receptor, leading to its activation and causing tumor migration and invasion in gliomas through the activation of an intracellular signal [33].

Hence, based on the role of MMP1 in glioma cells and its potential as a lectin target owing to glycan expression [34], we performed a docking in order to evaluate the interaction of ConGF with MMP1.

The docking scores of the ConGF and MMP1 *N*-glycans are represented in Table 1. ConGF was able to interact favorably with the MMP1 *N*-glycans, except N011, T004, and T013, which showed a lower score than Xman, considered as the reference ligand. In all cases, ConGF interacted with the mannosyl moieties present in the branches.

In the *N*-glycosylation process, carbohydrates are covalently attached to an asparagine residue in an Asn-X-Ser/Thr sequence, where X cannot be a proline. In eukaryotic cells, this process starts in the rough endoplasmic reticulum (RER) with the addition of a precursor glycan that is then modified in the Golgi apparatus (GA). The precursor consists of an oligosaccharide containing two residues of *N*-acetylglucosamine, nine of mannose, and three of glucose. This precursor can generate the three major types of *N*-glycans by the removal and addition of monosaccharides, such as *N*-acetylglucosamine, *N*-acetylgalactosamine, galactose, fucose, and sialic acid, thereby generating high-mannose, complex, and hybrid glycans [35,36,37,38].

**Figure 6:**
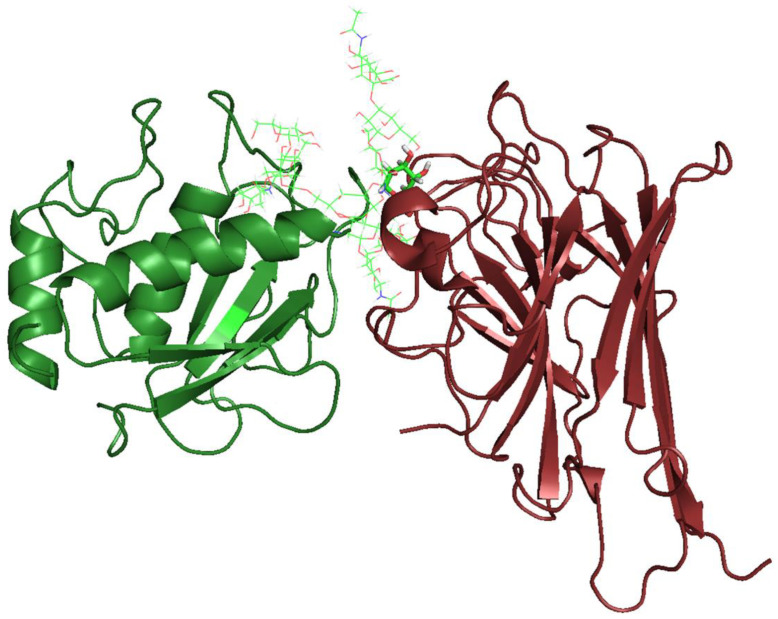
Interaction between ConGF (in red) and MMP1 catalytic domain (in green). Proteins are represented in cartoon and glycan N009 in green lines. The mannose residue interacting with ConGF CRD is highlighted in stick representation.

MMP1 has two potential glycosylation sites, Asn120 and Asn143, but only the glycosylation of Asn120 has been experimentally proven. This protein presents glycoforms that may range from 52 to 57 kDa to its molecular weight. The structures of their glycosylations were determined experimentally from fibroblast cells, which include a variety of complex-type biantennary glycans. In HT-1080 fibrosarcoma cells, the MMP1 glycosylation pattern was altered and became more heterogeneous with a predominance of biantennary glycans carrying Lewis X, LacdiNAc, sialylated LacdiNAc, and GalNAcβ1,4 (Fucα1,3) GlcNAc [34,39]. This change in the glycosylation pattern is a common event in cancer cells and is involved in many processes that may affect the survival and progression of tumors, as well as invasive and metastatic processes in most cancer cell lines, such as fibrosarcoma and glioma [39,40,41]. Our docking results indicate that ConGF interacts with the MMP1 catalytic domain via glycosylation and is capable of interacting with various glycoforms present in normal cells and malignant cells. A representation of the interaction between ConGF and MMP1 via N009 glycan, which demonstrated the highest docking score, is shown in Figure 6.

**Figure 7:**
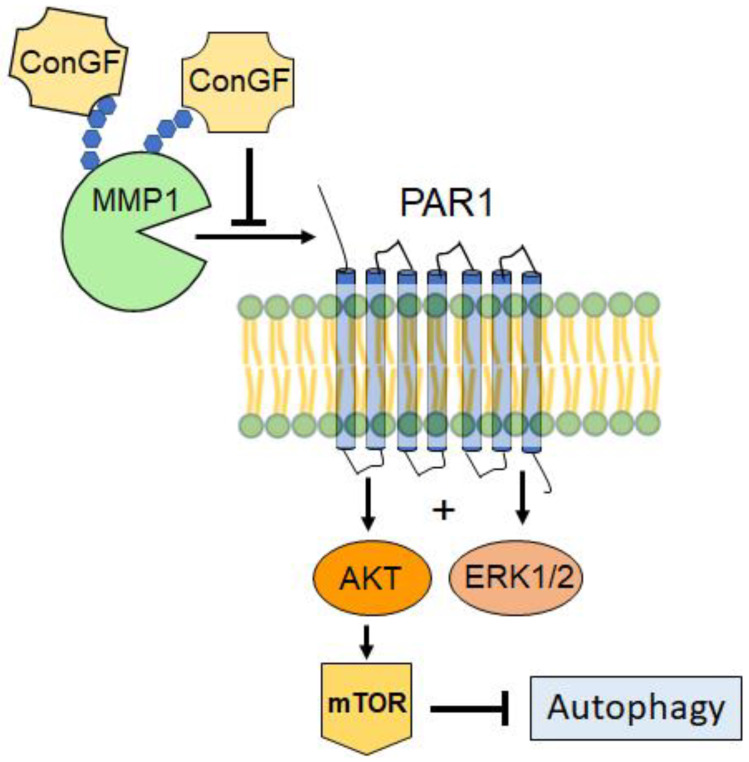
Postulated mechanism for ConGF activity via MMP1 interaction.

The overexpression of the matrix metalloproteinase-1 (MMP1) and proteinase-activated receptor-1 (PAR1) proteins have been correlated with the malignancy of gliomas and the KPS scores of patients, with a significantly shorter overall survival [25,33,42,43]. PAR1 is a member of a small family of seven membrane-spanning G-protein-coupled receptors (GPCRs) which are activated by proteolytic cleavage [44]. MMP-1 acts as a signaling molecule by cleaving a specific residue sequence within the *N*-terminal extracellular domain of PAR1, denoting a noncanonical mechanism of PAR1 activation [33,44,45,46]. Noteworthy, the downstream signaling pathways activated by PAR1 include MAPKs (ex. ERK1/2) and PI3K/AKT/mTOR [46] and may be associated with autophagy inhibition [47], increased proliferation, and migration [33], which may be important factors in the malignancy and invasion capacity of gliomas. Our study indicates that ConGF can interact with the glycans of MMP1 and trigger glioma cell death and autophagy. The mechanism for this process was not fully addressed, but one possibility could be the inhibition of MMP1 by ConGF, leading a downregulation of PAR1 and its downstream pathways, such as ERK1/2 and AKT/mTOR (Figure 7).

Noteworthy, other legume lectins have been described to be able to inhibit ERK1/2, AKT, and mTOR activity and stimulate autophagy [10,11,12]. Hence, our results are in line with previous studies with other legume lectins and present MMP1 as a possible target to trigger the main ConGF effects on glioma cells.

## 3. Methodology

### 3.1. Purification of Lectin ConGF

*Canavalia grandiflora* lectin (ConGF) was obtained according to the methods described by Ceccato (2010) [48]. ConGF was isolated by affinity chromatography on a Sephadex G-50, and its purity was assessed by polyacrylamide gel electrophoresis in the presence of SDS. The lectin was sterilely dissolved in glucose-free HEPES–saline buffer composed of 124 mM NaCl, 4 mM KCl, 1.2 mM MgSO_4_, 25 mM HEPES, and 1 mM CaCl_2_, pH 7.4, and used to prepare the concentrations to be tested by dilution in culture medium at the time of treatment. To verify the role of the lectin domain (CRD) in ConGF activity, this lectin was also sterilely dissolved in HEPES–saline buffer without glucose but containing 0.1 M of its specific binding sugar (α-methyl-d-mannoside) and kept for 30 min at 37 °C before being used for cell treatment.

### 3.2. Cell Culture of Rat Glioblastoma C6 and Treatment

C6 cells from Wistar rat glioblastoma (*Rattus norvegicus*) were acquired from the cell bank in Rio de Janeiro (Brazil). Cells were grown in culture bottles having 25 cm^2^ of growth area with Dulbecco’s Modified Eagle’s Medium (DMEM) supplemented with 10% (*v*/*v*) fetal bovine serum (FBS) (Gibco^®^, Ref. 12657, Grand Island, NY, USA), 100 units/mL penicillin, and 100 mg/mL streptomycin (Gibco^®^) at 37 °C in a humidified atmosphere of 95% air and 5% CO_2_. The medium was changed every two days until the cells reached 80% confluence. To carry out the experiments, cells were washed with phosphate-buffered saline (PBS) (140 mM NaCl, 3 mM KCl, 10 mM Na_2_HPO_4_, and 2 mM KH_2_PO_4_, pH 7.4) and chemically dissociated by trypsin. The cell pellet was resuspended with 3 mL of medium and the cell concentration determined by counting in a Neubauer chamber. After the 3rd passage, cells were used to carry out the experiments at the limit of use until the 12th passage. C6 cells were seeded in wells of 6-, 24-, or 96-well plates with DMEM medium supplemented with 10% (*v*/*v*) fetal bovine serum (FBS) (Gibco^®^), 100 units/mL penicillin, and 100 mg/mL streptomycin (Gibco^®^) for 24 h at 37 °C in a humidified atmosphere of 95% air and 5% CO_2_. Prior to addition of the lectin, the plates were observed under an inverted microscope to assess adherence and confluence. Hence, the culture media were replaced with fresh media containing vehicle or ConGF at concentrations of 10, 30, 50, or 100 μg/mL and incubated for 3, 6, 12, 24, or 48 h, depending on the time of treatment. The lectin was diluted with HEPES–saline buffer without glucose composed of NaCl 124 mM, KCl 4 mM, MgSO_4_ 1.2 mM, HEPES 25 mM, and CaCl_2_ 1 mM, pH 7.4. For all assays, the control cell cultures were incubated with vehicles (HEPES–saline buffer without glucose).

### 3.3. MTT Assay

Viability of cells after exposure to ConGF was evaluated by the colorimetric method with 3-[4,5-dimethylthiazol-2-yl]-2,5-diphenyltetrazolium (MTT). MTT is converted to a purple formazan insoluble after cleavage of the tetrazolium ring by cellular dehydrogenases. The purple formazan is proportional to cell viability [49]. For this assay, C6 cells were seeded in a 96-well plate at a density of 50,000 cells/mL, and upon completing the treatment period, the medium was removed, and cells were incubated for 1 h at 37 °C with 0.5 mg/mL MTT dissolved in HBSS (saline buffer containing 136 mM NaCl, 5.4 mM KCl, 1.4 mM MgCl_2_·6H_2_O, 1 mM NaH_2_PO_4_, 1.2 mM CaCl_2_·2H_2_O, 10 mM HEPES, and 9 mM glucose, pH 7.4). Reduced MTT formazan crystals were dissolved with 100 mL dimethylsulfoxide (DMSO) for 30 min at 37 °C, and the absorbance was evaluated at 540 nm using the Tecan^®^ (Tecan Group Ltd., Männedorf, Switzerland) Microplate Reader Infinite M200 located in the Laboratório Multiusuário de Estudos em Biologia at the Universidade Federal de Santa Catarina (LAMEB/UFSC). The results were expressed as a percentage of the control/vehicle group (considered as 100% viable). The values obtained through the absorbance reading were transformed into percentages of cell viability relative to the mean of the cell controls, considered as equivalent to 100% of viable cells. For this assay, 4 distinct cell growths (*n* = 4) were used and measured in triplicate.

### 3.4. Light Microscopy

For analysis of cell morphology, cells were seeded in wells of a 96-well plate at density of 100,000 cells/mL, and after 24 h, cells were incubated with vehicle (HEPES–saline buffer without glucose) or ConGF at concentrations of 10, 30, 50, or 100 μg/mL for 6, 12, 24, or 48 h. Cells were observed by light microscopy, where the images were captured with a digital camera coupled to an inverted microscope (Nikon Eclipse T2000-U, Melville, NY, USA).

### 3.5. Cell Migration (Scratch Assay)

C6 glioma cells were grown to confluence in a 24-well plate (10 × 10^4^ cells/well), and then a wound was introduced in each well by scraping cell layers with a P200 pipette tip. Cells were washed with PBS to remove those loosely held [50]. Thereafter, serum-free DMEM, containing vehicle (control) or ConGF, was added. Images were captured at the 0, 24, and 48 h treatment time points by an inverted NIKON eclipse T2000-U microscope. Percentage of wound closure was calculated using Image J software.

### 3.6. JC-1 Assay

Mitochondrial membrane potential (Δψm) was measured using 5,5′,6,6′-tetrachloro-1,1′,3,3′-tetraethyl-benzimidazolyl carbocyanine iodide (JC-1, Sigma-Aldrich, St. Louis, MO, USA), as described by Wolin et al. (2021) [12]. Briefly, C6 cells were seeded in a 96-well plate at 10^4^ cells/well for 24 h and then treated for 6 h with vehicle (control) or ConGF. After treatments, JC-1 solution (0.1 mM) was added, and cells were incubated for 20 min at 37 °C. Next, cells were washed with PBS (140 mM NaCl, 3 mM KCl, 10 mM Na_2_HPO_4_, and 2 mM KH_2_PO_4_, pH 7.4), followed by the addition of 0.1 mL/well PBS. Fluorescence was measured in a Spectramax Paradigm Microplate Reader (Molecular Devices^®^, Sunnyvale, CA, USA) set at 490 nm excitation and 520 nm emission for detection of red fluorescence (JC-1 aggregates) and 525 nm excitation and 590 nm emission for green detection (JC-1 monomers). The values of red/green fluorescence ratio of each sample were converted to percentages relative control/vehicle-treated cells. Carbonyl cyanide 4-(trifluoromethoxy) phenylhydrazone (FCCP, 1 μM; Sigma^®^) was used as a positive control for mitochondrial depolarization. The assays were performed in 4 independent experiments in triplicate.

### 3.7. Propidium Iodide (PI) Staining

To characterize cell death, a double-labeling with Hoechst and propidium iodide (PI) was used [12]. The fluorescent nuclear marker PI is a probe used to evidence cell death by necrosis or later apoptosis, as it is able to penetrate cells only with compromised plasma membrane, emitting red fluorescence. Hoechst is a fluorescent marker that penetrates cells with intact or ruptured membranes, binding to DNA and emitting blue fluorescence. Hence, 96-well plates were seeded at a density of 10 × 10^4^ cells per well, and after 24 h, culture medium was removed, and cells were treated with vehicle or ConGF (10, 30, 50, or 100 μg/mL) for 24 or 48 h. Cells were washed with PBS and incubated for 15 min in the dark with binding buffer (composed of 0.01 M HEPES (pH 7.4), 140 mM of NaCl, and 25 mM CaCl_2_), containing Hoechst (1 μg/mL; Sigma-Aldrich, St. Louis, MO, USA) and PI (14 μg/mL Sigma-Aldrich, St. Louis, MO, USA). Then, cells were analyzed by fluorescence microscopy (Nikon Eclipse T2000-U), using filter sets, 488 nm excitation and 560 nm emission for PI and 353 nm excitation and 483 nm emission for Hoechst. The captured images were analyzed using ImageJ software, and fluorescence was quantified. Four independent experiments were carried out in triplicate. The captured images were analyzed using ImageJ software, and fluorescence was quantified.

### 3.8. Acridine Orange Staining of Acidic Vesicular Organelle

Acridine Orange (AO) assay was performed to evaluate acidic vesicular organelle (AVO) formation by ConGF, as described by Wolin et al. (2021) [12]. Briefly, C6 cells were seeded in a 96-well plate at 10 × 10^4^ cells/well for 24 h in DMEM. Then, C6 cells were treated with vehicle (control) or ConGF at 10 or 30 μg/mL for 24 or 48 h. Thereafter, 20 μL of AO (10 μg/mL; Sigma^®^) were added for 20 min in the dark at 37 °C. Cells were visualized by an inverted NIKON eclipse T2000-U microscope, using filter sets of 470 nm excitation and 525 nm emission for chromatin (CR; green fluorescence) and 350 nm excitation and 615 nm emission for acidic vesicular organelles (AVO; orange/red fluorescence) detection.

### 3.9. Western Blot

C6 cells were seeded in a 6-well plate at a density of 25 × 10^4^ cells per well for 24 h. Then, cells were treated with vehicle (control) or ConGF (10 or 30 μg/mL) in serum-free DMEM for 12 h. At the end of treatments, cells were homogenized in 200 μL Stop Solution (Tris 50 mM, EDTA 2 mM, SDS 4%, pH 6.8), as previously described by Nascimento et al. (2018) [10]. Protein extracts (30 μg/sample) were electrophoresed in 12% SDS-PAGE minigels and transferred onto nitrocellulose membranes using a semi-dry blotting apparatus (1.2 mA/cm^2^ by 1.5 h). Transfer efficiency was confirmed by Ponceau S staining of membranes. The immunodetection of LC3ABI (16 kDa) and LC3ABII (14 kDa) was performed by overnight incubation with the primary antibody (Cell Signaling; 1:1000) diluted in Tris-buffered saline Tween 20 (TBS-T) containing 2% BSA. β-actin detection (Santa Cruz Biotechnology, Dallas, Texas U.S.A.; 1:2500 dilution) was used as the loading control. The membranes were washed in TBS-T and incubated for 1 h at room temperature with horseradish peroxidase (HRP)-conjugated anti-IgG-rabbit antibodies. The bands were developed by chemiluminescence substrate (Super ECL, GE**^®^,** Waltham, MA, USA) on ChemiDoc Imaging System (Bio-Rad, Berkeley, CA, USA) equipment located in the Laboratório Multiusuário de Estudos em Biologia, Universidade Federal de Santa Catarina (LAMEB/UFSC). Bands and OD were captured and quantified using Image Lab^®^ software (Bio-Rad, Berkeley, CA, USA). Measurement of LC3 was performed by dividing the OD of each form of LC3 (I and II) by the OD of β-actin. Hence, in order to express the ratio of LC3, normalized OD of LC3II (conjugated with phosphatidylethanolamine and present in the autophagosome vesicles) was divided by normalized OD of LC3I (cytosolic).

### 3.10. Molecular Docking

ConGF three-dimensional structure (PDB id: 4L8Q) [15] was subjected to molecular docking with *N*-glycans using GOLD v. 5.5 (CDCC, Cambridge, UK). This program tests the interaction of proteins with ligands, allowing a total ligand flexibilization and a partial flexibilization of protein residues [51,52]. Parameters used included population size of 100, number of operations of 10,000, selection pressure of 1.1, number of islands of 5, niche size of 2, crossover frequency of 95, number of poses of 40, and PLANTSPLP score function [53]. The CRD cavity was chosen as the region of analysis with all amino acid residues and 2 structural water molecules in a 10 Å radius. Better interaction models were chosen by evaluating the score, hydrogen and hydrophobic interactions, geometric penalties of the ligand, and comparison with data obtained from redocking with Xman, a ligand present in the crystallographic structure [15,51,54]. Glycan structures were generated and energetically minimized through the Carbohydrate Builder server (Woods Group, Glycan Web, University of Georgia at Athens, GA, USA, http://glycam.org, accessed on 15 April 2022) and GLYCAM_06 force field [55], respectively. These were designed based on the structural information of MMP1 glycosylation previously studied [34]. The tested *N*-glycans are schematically illustrated in Supplementary Figures S1 and S2. ConGF-MMP1 protein–protein docking was performed using the Patchdock server [56] in which the built algorithm aims to find interaction sites between two molecules by the identification of geometric complementarity regions. The generated solutions are ranked by shape complementarity and atomic contact energy. ConGF was defined as the receptor file and MMP1 as the ligand molecule. The settings were set to default and clustered RMSD to 4.0 Å. A file containing the residues of ConGF CRD was also uploaded to the server in order to limit the search to this region. PyMol (Schrodinger LLC, New York, NY, USA) was used to analyze data and generate figures.

## 4. Conclusions

In the present work, the antitumor potential of ConGF on the C6 glioma cells model was evaluated. The treatment showed that ConGF promoted a significant cytotoxic effect in the treated cells and was able to induce morphological changes and reduce cell density, in addition to inducing cell death by an autophagic mechanism. This effect is accompanied by a mitochondrial change at an early period and dependent on the carbohydrate recognition domain of the lectin. Furthermore, ConGF was able to decrease the cell migration at higher lectin concentrations. Furthermore, ConGF was able to favorably interact with glycans present in MMP1, which may be related to that elicited by this lectin. MMP1 could be an important element for signal modulation through PAR1 and thus induce death. In general, the data presented in this work indicate that ConGF has a significant ability to induce autophagy on C6 glioma cells, which may have applicability in antiglioma therapy.

## Figures and Tables

**Table 1:** Interactions of ConGF with Xman and MMP1 *N*-glycans.

Carbohydrate	Score
Xman	−45.26
MMP1 *N*-glycans in normal cells	
Glycan	Score
N001	−64.29
N002	−55.18
N003	−51.11
N004	−58.72
N005	−63.28
N006	−52.70
N007	−50.25
N008	−64.64
N009	−64.68
N010	−49.00
N011	-
MMP1 *N*-glycans in malignant cells	
Glycan	Score
T001	−50.62
T002	−63.96
T003	−57.95
T004	-
T005	−59.12
T006	−47.63
T007	−52.33
T008	−60.83
T009	−53.75
T010	−59.61
T011	−45.05
T012	−44.67
T013	-
T014	−54.20
T015	−57.97
T016	−47.46
T017	−53.89

## Data Availability

Data available upon request to the authors.

## Supplementary Materials

The following supporting information can be downloaded at: https://www.mdpi.com/article/10.3390/molecules27207089/s1, Supplementary Figure S1: MMP1 N-glycan structures present in normal fibroblast cells. Representations based on the experimental results of Saarinen et al., 1999; Supplementary Figure S2: MMP1 N-glycan structures present in HT-1080 fibrosarcoma cells. Representations based on the experimental results of Saarinen et al., 1999.

## References

[1] Horbinski C., Berger T., Packer R.J., Wen P.Y. (2022). Clinical Implications of the 2021 Edition of the WHO Classification of Central Nervous System Tumours. Nat. Rev. Neurol..

[2] Tucker-Burden C., Chappa P., Krishnamoorthy M., Gerwe B.A., Scharer C.D., Heimburg-Molinaro J., Harris W., Usta S.N., Eilertson C.D., Hadjipanayis C.G. (2012). Lectins Identify Glycan Biomarkers on Glioblastoma-Derived Cancer Stem Cells. Stem Cells Dev..

[3] Louis D.N., Perry A., Reifenberger G., von Deimling A., Figarella-Branger D., Cavenee W.K., Ohgaki H., Wiestler O.D., Kleihues P., Ellison D.W. (2016). The 2016 World Health Organization Classification of Tumors of the Central Nervous System: A Summary. Acta Neuropathol..

[4] Guidotti G., Brambilla L., Rossi D. (2020). Exploring Novel Molecular Targets for the Treatment of High-Grade Astrocytomas Using Peptide Therapeutics: An Overview. Cells.

[5] Mazalovska M., Kouokam J.C. (2020). Plant-Derived Lectins as Potential Cancer Therapeutics and Diagnostic Tools. Biomed. Res. Int..

[6] De Mejía E.G., Prisecaru V.I. (2005). Lectins as Bioactive Plant Proteins: A Potential in Cancer Treatment. Crit. Rev. Food Sci. Nutr..

[7] Yau T., Dan X., Ng C.C.W., Ng T.B. (2015). Lectins with Potential for Anti-Cancer Therapy. Molecules.

[8] Cavada B.S., Pinto-Junior V.R., Osterne V.J.S., Nascimento K.S. (2018). ConA-Like Lectins: High Similarity Proteins as Models to Study Structure/Biological Activities Relationships. Int. J. Mol. Sci..

[9] Pratt J., Annabi B. (2014). Induction of Autophagy Biomarker BNIP3 Requires a JAK2/STAT3 and MT1-MMP Signaling Interplay in Concanavalin-A-Activated U87 Glioblastoma Cells. Cell. Signal..

[10] Nascimento A.P.M., Knaut J.L., Rieger D.K., Wolin I.A.V., Heinrich I.A., Mann J., Juarez A.V., del Sosa L.V., De Paul A.L., Moreira C.G. (2018). Anti-Glioma Properties of DVL, a Lectin Purified from Dioclea Violacea. Int. J. Biol. Macromol..

[11] Nascimento A.P.M., Wolin I.A.V., Welter P.G., Heinrich I.A., Zanotto-Filho A., Osterne V.J.S., Lossio C.F., Silva M.T.L., Nascimento K.S., Cavada B.S. (2019). Lectin from Dioclea Violacea Induces Autophagy in U87 Glioma Cells. Int. J. Biol. Macromol..

[12] Wolin I.A.V., Heinrich I.A., Nascimento A.P.M., Welter P.G., Sosa L.D.V., De Paul A.L., Zanotto-Filho A., Nedel C.B., Lima L.D., Osterne V.J.S. (2021). ConBr Lectin Modulates MAPKs and Akt Pathways and Triggers Autophagic Glioma Cell Death by a Mechanism Dependent upon Caspase-8 Activation. Biochimie.

[13] Quintero-Fabián S., Arreola R., Becerril-Villanueva E., Torres-Romero J.C., Arana-Argáez V., Lara-Riegos J., Ramírez-Camacho M.A., Alvarez-Sánchez M.E. (2019). Role of Matrix Metalloproteinases in Angiogenesis and Cancer. Front. Oncol..

[14] Sounni N.E., Devy L., Hajitou A., Frankenne F., Munaut C., Gilles C., Deroanne C., Thompson E.W., Foidart J.M., Noel A. (2002). MT1-MMP Expression Promotes Tumor Growth and Angiogenesis through an up-Regulation of Vascular Endothelial Growth Factor Expression. FASEB J..

[15] Barroso-Neto I.L., Simões R.C., Rocha B.A.M., Bezerra M.J.B., Pereira-Junior F.N., Silva Osterne V.J., Nascimento K.S., Nagano C.S., Delatorre P., Pereira M.G. (2014). Vasorelaxant Activity of Canavalia Grandiflora Seed Lectin: A Structural Analysis. Arch. Biochem. Biophys..

[16] Nunes B.S., Rensonnet N.S., Dal-Secco D., Vieira S.M., Cavada B.S., Teixeira E.H., Moura T.R., Teixeira C.S., Clemente-Napimoga J.T., Cunha F.Q. (2009). Lectin Extracted from Canavalia Grandiflora Seeds Presents Potential Anti-Inflammatory and Analgesic Effects. Naunyn Schmiedebergs Arch. Pharmacol..

[17] Ly J.D., Grubb D.R., Lawen A. (2003). The Mitochondrial Membrane Potential (deltapsi(m)) in Apoptosis: An Update. Apoptosis.

[18] Thomé M.P., Filippi-Chiela E.C., Villodre E.S., Migliavaca C.B., Onzi G.R., Felipe K.B., Lenz G. (2016). Ratiometric Analysis of Acridine Orange Staining in the Study of Acidic Organelles and Autophagy. J. Cell Sci..

[19] Chang C.-P., Yang M.-C., Liu H.-S., Lin Y.-S., Lei H.-Y. (2007). Concanavalin A Induces Autophagy in Hepatoma Cells and Has a Therapeutic Effect in a Murine in Situ Hepatoma Model. Hepatology.

[20] Roy B., Pattanaik A.K., Das J., Bhutia S.K., Behera B., Singh P., Maiti T.K. (2014). Role of PI3K/Akt/mTOR and MEK/ERK Pathway in Concanavalin A Induced Autophagy in HeLa Cells. Chem. Biol. Interact..

[21] Bhutia S.K., Panda P.K., Sinha N., Praharaj P.P., Bhol C.S., Panigrahi D.P., Mahapatra K.K., Saha S., Patra S., Mishra S.R. (2019). Plant Lectins in Cancer Therapeutics: Targeting Apoptosis and Autophagy-Dependent Cell Death. Pharmacol. Res..

[22] Leal R.B., Pinto-Junior V.R., Osterne V.J.S., Wolin I.A.V., Nascimento A.P.M., Neco A.H.B., Araripe D.A., Welter P.G., Neto C.C., Correia J.L.A. (2018). Crystal Structure of DlyL, a Mannose-Specific Lectin from Dioclea Lasiophylla Mart. Ex Benth Seeds That Display Cytotoxic Effects against C6 Glioma Cells. Int. J. Biol. Macromol..

[23] Hagemann C., Anacker J., Ernestus R.-I., Vince G.H. (2012). A Complete Compilation of Matrix Metalloproteinase Expression in Human Malignant Gliomas. World J. Clin. Oncol..

[24] Cui N., Hu M., Khalil R.A. (2017). Biochemical and Biological Attributes of Matrix Metalloproteinases. Prog. Mol. Biol. Transl. Sci..

[25] Stojic J., Hagemann C., Haas S., Herbold C., Kühnel S., Gerngras S., Roggendorf W., Roosen K., Vince G.H. (2008). Expression of Matrix Metalloproteinases MMP-1, MMP-11 and MMP-19 Is Correlated with the WHO-Grading of Human Malignant Gliomas. Neurosci. Res..

[26] Nakagawa T., Kubota T., Kabuto M., Sato K., Kawano H., Hayakawa T., Okada Y. (1994). Production of Matrix Metalloproteinases and Tissue Inhibitor of Metalloproteinases-1 by Human Brain Tumors. J. Neurosurg..

[27] McCready J., Broaddus W.C., Sykes V., Fillmore H.L. (2005). Association of a Single Nucleotide Polymorphism in the Matrix Metalloproteinase-1 Promoter with Glioblastoma. Int. J. Cancer.

[28] Ruhul Amin A.R.M., Oo M.L., Senga T., Suzuki N., Feng G.-S., Hamaguchi M. (2003). SH2 Domain Containing Protein Tyrosine Phosphatase 2 Regulates Concanavalin A-Dependent Secretion and Activation of Matrix Metalloproteinase 2 via the Extracellular Signal-Regulated Kinase and p38 Pathways. Cancer Res..

[29] Gingras D., Pagé M., Annabi B., Béliveau R. (2000). Rapid Activation of Matrix Metalloproteinase-2 by Glioma Cells Occurs through a Posttranslational MT1-MMP-Dependent Mechanism. Biochim. Biophys. Acta.

[30] Sina A., Proulx-Bonneau S., Roy A., Poliquin L., Cao J., Annabi B. (2010). The Lectin Concanavalin-A Signals MT1-MMP Catalytic Independent Induction of COX-2 through an IKKgamma/NF-kappaB-Dependent Pathway. J. Cell Commun. Signal..

[31] Pratt J., Roy R., Annabi B. (2012). Concanavalin-A-Induced Autophagy Biomarkers Requires Membrane Type-1 Matrix Metalloproteinase Intracellular Signaling in Glioblastoma Cells. Glycobiology.

[32] Anand M., Van Meter T.E., Fillmore H.L. (2011). Epidermal Growth Factor Induces Matrix Metalloproteinase-1 (MMP-1) Expression and Invasion in Glioma Cell Lines via the MAPK Pathway. J. Neurooncol..

[33] Zhang Y., Zhan H., Xu W., Yuan Z., Lu P., Zhan L., Li Q. (2011). Upregulation of Matrix Metalloproteinase-1 and Proteinase-Activated Receptor-1 Promotes the Progression of Human Gliomas. Pathol. Res. Pract..

[34] Saarinen J., Welgus H.G., Flizar C.A., Kalkkinen N., Helin J. (1999). N-Glycan Structures of Matrix Metalloproteinase-1 Derived from Human Fibroblasts and from HT-1080 Fibrosarcoma Cells. Eur. J. Biochem..

[35] Ghazarian H., Idoni B., Oppenheimer S.B. (2011). A Glycobiology Review: Carbohydrates, Lectins and Implications in Cancer Therapeutics. Acta Histochem..

[36] Nagae M., Yamaguchi Y. (2012). Function and 3D Structure of the N-Glycans on Glycoproteins. Int. J. Mol. Sci..

[37] Pinho S.S., Reis C.A. (2015). Glycosylation in Cancer: Mechanisms and Clinical Implications. Nat. Rev. Cancer.

[38] Bieberich E. (2014). Synthesis, Processing, and Function of N-Glycans in N-Glycoproteins. Adv. Neurobiol..

[39] Boon L., Ugarte-Berzal E., Vandooren J., Opdenakker G. (2016). Glycosylation of Matrix Metalloproteases and Tissue Inhibitors: Present State, Challenges and Opportunities. Biochem. J..

[40] Yamamoto H., Swoger J., Greene S., Saito T., Hurh J., Sweeley C., Leestma J., Mkrdichian E., Cerullo L., Nishikawa A. (2000). Beta1,6-N-Acetylglucosamine-Bearing N-Glycans in Human Gliomas: Implications for a Role in Regulating Invasivity. Cancer Res..

[41] Stowell S.R., Ju T., Cummings R.D. (2015). Protein Glycosylation in Cancer. Annu. Rev. Pathol..

[42] Pullen N.A., Anand M., Cooper P.S., Fillmore H.L. (2012). Matrix Metalloproteinase-1 Expression Enhances Tumorigenicity as Well as Tumor-Related Angiogenesis and Is Inversely Associated with TIMP-4 Expression in a Model of Glioblastoma. J. Neurooncol..

[43] Xu Y., Zhong Z., Yuan J., Zhang Z., Wei Q., Song W., Chen H. (2013). Collaborative Overexpression of Matrix Metalloproteinase-1 and Vascular Endothelial Growth Factor-C Predicts Adverse Prognosis in Patients with Gliomas. Cancer Epidemiol..

[44] Austin K.M., Covic L., Kuliopulos A. (2013). Matrix Metalloproteases and PAR1 Activation. Blood.

[45] Goerge T., Barg A., Schnaeker E.-M., Poppelmann B., Shpacovitch V., Rattenholl A., Maaser C., Luger T.A., Steinhoff M., Schneider S.W. (2006). Tumor-Derived Matrix Metalloproteinase-1 Targets Endothelial Proteinase-Activated Receptor 1 Promoting Endothelial Cell Activation. Cancer Res..

[46] Tomko N., Kluever M., Wu C., Zhu J., Wang Y., Salomon R.G. (2020). 4-Hydroxy-7-Oxo-5-Heptenoic Acid Lactone Is a Potent Inducer of Brain Cancer Cell Invasiveness That May Contribute to the Failure of Anti-Angiogenic Therapies. Free Radic. Biol. Med..

[47] Bode M.F., Schmedes C.M., Egnatz G.J., Bharathi V., Hisada Y.M., Martinez D., Kawano T., Weithauser A., Rosenfeldt L., Rauch U. (2021). Cell Type-Specific Roles of PAR1 in Coxsackievirus B3 Infection. Sci. Rep..

[48] Ceccatto V.M., Cavada B.S., Nunes E.P., Nogueira N.A.P., Grangeiro M.B., Moreno F.B.M.B., Teixeira E.H., Sampaio A.H., Alves M.A.O., Ramos M.V. (2002). Purification and Partial Characterization of a Lectin from Canavalia Grandiflora Benth. Seeds. Protein Pept. Lett..

[49] Mosmann T. (1983). Rapid Colorimetric Assay for Cellular Growth and Survival: Application to Proliferation and Cytotoxicity Assays. J. Immunol. Methods.

[50] Liang C.-C., Park A.Y., Guan J.-L. (2007). In Vitro Scratch Assay: A Convenient and Inexpensive Method for Analysis of Cell Migration in Vitro. Nat. Protoc..

[51] De Ávila M.B., Xavier M.M., Pintro V.O., de Azevedo W.F. (2017). Supervised Machine Learning Techniques to Predict Binding Affinity. A Study for Cyclin-Dependent Kinase 2. Biochem. Biophys. Res. Commun..

[52] Jones G., Willett P., Glen R.C., Leach A.R., Taylor R. (1997). Development and Validation of a Genetic Algorithm for Flexible Docking. J. Mol. Biol..

[53] Korb O., Stützle T., Exner T.E. (2009). Empirical Scoring Functions for Advanced Protein-Ligand Docking with PLANTS. J. Chem. Inf. Model..

[54] Heck G.S., Pintro V.O., Pereira R.R., de Ávila M.B., Levin N.M.B., de Azevedo W.F. (2017). Supervised Machine Learning Methods Applied to Predict Ligand- Binding Affinity. Curr. Med. Chem..

[55] Kirschner K.N., Yongye A.B., Tschampel S.M., González-Outeiriño J., Daniels C.R., Foley B.L., Woods R.J. (2008). GLYCAM06: A Generalizable Biomolecular Force Field. Carbohydrates. J. Comput. Chem..

[56] Schneidman-Duhovny D., Inbar Y., Nussinov R., Wolfson H.J. (2005). PatchDock and SymmDock: Servers for Rigid and Symmetric Docking. Nucleic Acids Res..

