# Supplementary material for: Structural Prediction and Characterization of Canavalia grandiflora (ConGF) Lectin Complexed with MMP1: Unveiling the Antiglioma Potential of Legume Lectins
Source: Molecules. 2022 Oct 20;27(20):7089. doi: 10.3390/molecules27207089 (PMC9612022; doi:10.3390/molecules27207089)
Supplement: Supplementary file 1 [file molecules-27-07089-s001.zip › molecules-1956257-supplementary.pdf]

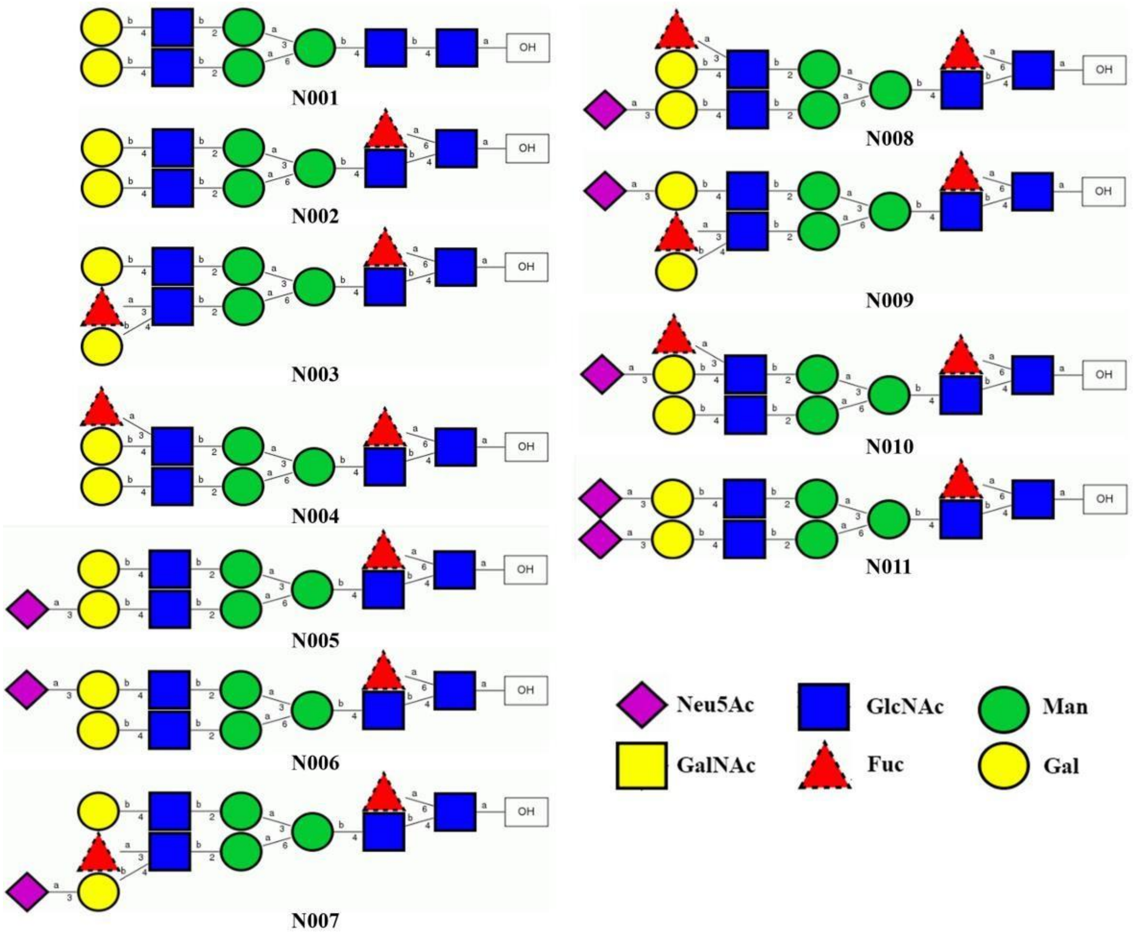

Figure S1. MMP1 *N*-glycan structures present in normal fibroblast cells. Representations based on the experimental results of Saarinen et al., 1999.

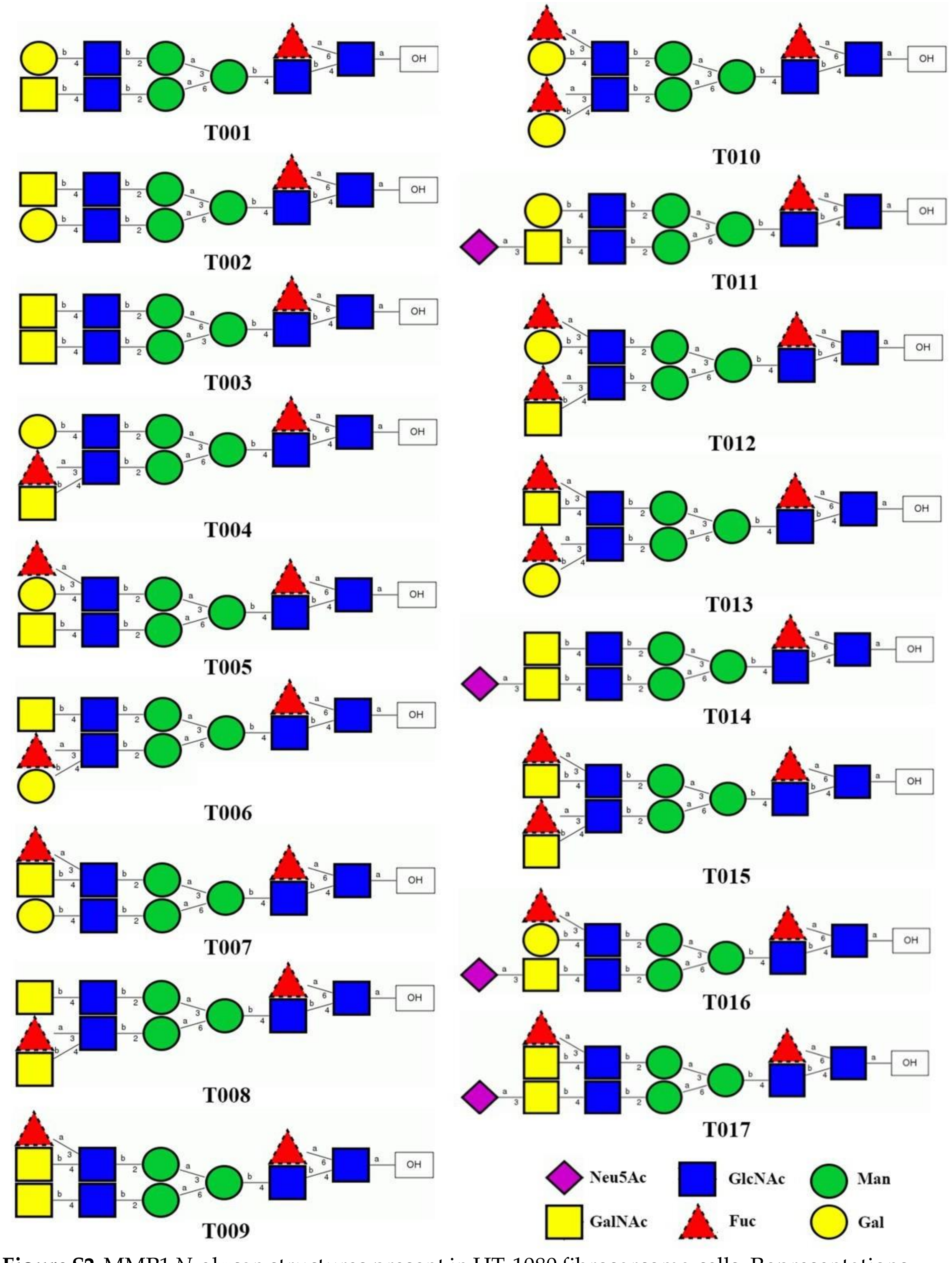

Figure S2. MMP1 *N*-glycan structures present in HT-1080 fibrosarcoma cells. Representations based on the experimental results of Saarinen et al., 1999.
